# A novel cell immunoassay to measure survival of motor neurons protein in blood cells

**DOI:** 10.1186/1471-2377-6-6

**Published:** 2006-02-01

**Authors:** Stephen J Kolb, Amelie K Gubitz, Robert F Olszewski, Elizabeth Ottinger, Charlotte J Sumner, Kenneth H Fischbeck, Gideon Dreyfuss

**Affiliations:** 1 Howard Hughes Medical Institute And Department of Biochemistry & Biophysics, University of Pennsylvania School of Medicine, Philadelphia, Pennsylvania, 19104-6148, USA; 2 Neurogenetics Branch, National Institute of Neurological Disorders and Stroke, Bethesda, MD 20892, USA

## Abstract

**Background:**

The motor neuron degenerative disease spinal muscular atrophy (SMA) is the leading genetic cause of infant mortality and is caused by mutations in the survival of motor neurons (SMN) gene that reduce the expression levels of the SMN protein. A major goal of current therapeutic approaches is to increase SMN levels in SMA patients. The purpose of this study was to develop a reliable assay to measure SMN protein levels from peripheral blood samples.

**Methods:**

We developed a novel cell immunoassay to quantitatively measure SMN levels from peripheral blood mononuclear cells (PBMCs) using a single anti-SMN antibody.

**Results:**

SMN levels determined by the cell immunoassay are comparable to levels determined by Western blot, but in contrast, the immunoassay does not involve cell lysis, requires a small amount of patient material, and can be done on a large number of samples simultaneously. SMN levels from PBMCs are not influenced by cell type heterogeneity.

**Conclusion:**

SMN levels measured from total PBMCs provide an important snapshot of SMN protein expression, which should be a useful aid in SMA diagnosis, and a surrogate marker of efficacy of treatment in SMA clinical trials.

## Background

Spinal muscular atrophy (SMA) is one of the most common autosomal recessive diseases, affecting approximately 1 in 6,000 to 10,000 live births, and is the leading hereditary cause of infant mortality [1,2]. SMA is a neurodegenerative disease of motor neurons that results in progressive muscle weakness and death from respiratory failure, and is caused by mutations in the survival of motor neurons (SMN) gene [3]. These mutations result in a reduction in SMN protein expression and several studies have shown that SMN protein levels are reduced in cell lines and tissues derived from type I SMA patients compared to controls [4-7]. An obvious therapeutic strategy for this disease is, therefore, to attempt to increase SMN expression levels.

In recent years, several drugs have been shown to increase SMN mRNA and/or protein levels in cultured cells [8-16]. As a result, many of these compounds currently are, or are planned to be, in clinical trials. The design of therapeutic clinical trials for SMA patients hinges upon the expectation that survival or objective improvement in phenotype will be achieved. These benefits, if they are to occur, are long-term outcomes [17]. Thus, there is a need for biochemical surrogate assays to determine whether SMN levels are affected in patients that receive such treatments. SMN mRNA has been measured in blood by quantitative RT-PCR in a small number of patients in one previous study [18], however RT-PCR requires amplification of patient material and mRNA levels do not necessarily correlate with the amount of protein expressed in the cells.

We have developed a cell immunoassay to measure SMN levels in peripheral blood samples. We further compare this assay with the standard Western blot method for quantifying SMN protein and show that this method is feasible for use with patient blood samples during clinical trials.

## Methods

### Cell lines

Lymphoblastoid cell lines GM12497 (derived from a 7 month old control patient) and GM10684 (derived from a 6 month old type I SMA patient) were purchased from Coriell Cell Repositories (New Jersey) and maintained in RPMI media with 10% fetal bovine serum.

### Subjects and blood draws

Blood samples from anonymous, control individuals were collected in the department of transfusion medicine at the NIH as part of an NIH IRB approved protocol. Peripheral blood mononuclear cells (PBMCs) were isolated by standard Ficoll density gradient centrifugation, resuspended in fetal bovine serum with 10% DMSO and were cryopreserved. Pooled monocytes and lymphocytes were also obtained from the department of transfusion medicine at the NIH. Cells were then sent by overnight courier to the University of Pennsylvania on dry ice for protein level determination.

### Western blot

The anti-SMN (62E7) and anti-Y14 (1F12) monoclonal antibodies have been described previously [19,20]. The anti-mouse IgG secondary antibody labeled with IRDye ^®^800 (Rockland) was used at 1:5000. Proteins from 20 μg of total extracts were separated on NuPAGE^® ^4–12% Bis-Tris gels (Invitrogen) and transferred to nitrocellulose membranes. Quantitative immunoblotting was performed as suggested by the manufacturer (Li-Cor). The membranes were scanned on an Odyssey^® ^infrared imaging system (Li-Cor), and the intensity of the protein bands was analyzed using the software provided by the manufacturer.

### Surrogate SMN level cell immunoassay

Cell immunoassays to determine SMN protein levels were performed using black, clear-bottomed 96-well plates pre-coated with poly-ornithine (Sigma M0562). Immediately before the assay, cryopreserved PBMC samples were thawed, washed in phosphate-buffered saline (PBS) and counted with an automated NucleoCounter cell counter (New Brunswick Scientific). 1.5 × 10^5 ^cells/well were adhered to the well bottom by centrifugation (700 × g) for 5 minutes at room temperature. The cells were then fixed to the plate with 2.5% formaldehyde for 30 min and washed in PBS. All washes were performed with PBS using a Biotek ELX405 automatic plate washer. Cells were permeabilized with 0.1% Triton for 5 min and blocked with 20% FBS for 1 hour. The antibody used for the immunosorbant assay was monoclonal antibody 2B1 (1:500) against SMN. The plates were washed, and bound antibodies were detected using peroxidase-conjugated goat anti-mouse IgG (Jackson ImmunoResearch) and Supersignal^® ^ELISA Femto Maximum Sensitivity Substrate (Pierce). The luminescent intensity within each well was measured using a Perkin Elmer Victor2 plate reader. Signal background was determined for each sample by omitting the primary antibody.

### Statistical analysis

All error bars are standard deviation from the mean. The cell number titration data was fitted to a sigmoid curve using IGor.

## Results and discussion

**Figure 1:**
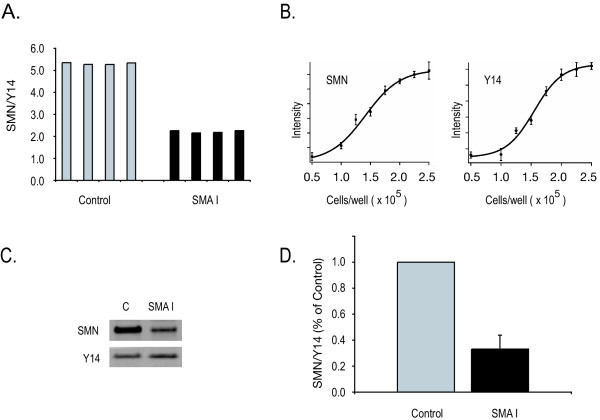
A) SMN levels in control and SMA I lymphoblastoid cell lines from four separate experiments. B) SMN and Y14 chemiluminescent intensity levels as a function of number of cells loaded per 96-well plate well. C) SMN levels from the same cell lines determined by Western blot. D) Quantification of Western blot data N = 9. Error bars are standard deviation from the mean.

We have developed a simple cell immunoassay to determine SMN protein levels that can be performed in 96-well plates and potentially higher density formats. To assess the influence of various parameters, we used a lymphoblastoid cell line derived from a type I SMA patient (GM10684) and a similarly established cell line from an age- and gender-matched individual as a control (GM12497) that we have previously characterized [21]. For each experiment, the common exon-junction complex component protein Y14 was also determined to confirm equal loading of wells. As expected, SMN levels in patient cells were significantly reduced compared to control (Figure 1A). When expressed as a ratio of SMN to Y14 expression, control SMN levels were 5.31 ± 0.04 and SMA I levels were 2.21 ± 0.05(N = 4). The optimal number of patient lymphoblastoid cells to load into a 96-well plate well, determined for this assay, was 1.5 × 10^5 ^cells (Figure 1B). Immunoblotting performed at the same time confirmed that SMN levels in the patient cells were reduced (Figure 1C and 1D). The degree of reduction in SMN protein measured by the 96-well plate-based assay (58.3 ± 7.0%, N = 4) was comparable to that measured by Western blot (66.8 ± 10.7%, N = 9).

**Figure 2:**
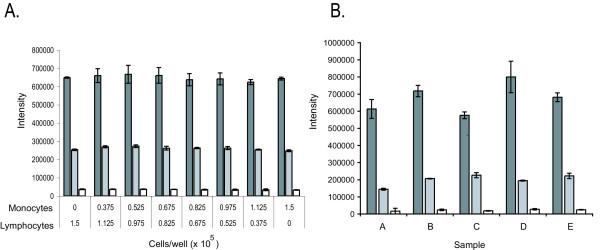
SMN (dark grey bars), Y14 (light grey bars) and background (white bars) chemiluminescent intensity levels in (A) sorted monocytes and lymphocytes, including mixed ratios of the two cell types, and in (B) peripheral blood mononuclear cell isolated from blood obtained from five control individuals. Error bars are standard deviation from the mean, N = 3.

Blood samples are the most practical source of patient cells for SMN protein level determination and can be measured repeatedly over time. We chose to study the peripheral blood mononuclear cells (PBMC) because they are readily isolated and typically represent the physiological cellular environment. One potential source of variability of using the PBMC is cell type heterogeneity, which can vary between different individuals and within the same individual at different times. We therefore compared SMN levels in pure populations of human monocytes and lymphocytes and found no significant difference when the same number of cells was analyzed (Figure 2A). Moreover, mixing the cell types had no effect on the SMN or Y14 levels. SMN levels from peripheral blood of control individuals are shown in Figure 2B. The intensity values for SMN and Y14 in PBMCs are similar in the pure and mixed monocyte and lymphocyte populations.

The SMN protein cell immunoassay compares SMN expression in an equivalent number of PBMC cells and does not depend on a stable endogenous control. Nonetheless, the use of Y14 as a stable endogenous control in these experiments, as would be done for Western blot analysis, lends further confidence in the assay to detect specific changes in SMN expression between two well-characterized cell lines. The SMN protein cell immunoassay method does not require cell lysis as do western blotting and sandwich ELISA methods. Moreover, there is no transfer of protein to a membrane and a relatively small amount of biological sample is required. We find that SMN levels can be measured in triplicate from a 5 to 8 ml sample of peripheral blood.

Nucleated cells from blood were chosen for protein determinations because the SMN protein is found both in the cytoplasm and the nucleus of cells. The measurement of SMN in the PBMC is valid despite the fact that the cell type composition of the PBMC can vary between individuals and even within the same individual at different times. We addressed the issue of cell type heterogeneity by measuring SMN in pure populations of PBMC components and found no effect of cell type composition. Recently, we also described a reliable assay to measure a well established function of the SMN protein, small nuclear ribonucleoprotein (snRNP) assembly [21,22]. In combination, the SMN protein cell immunoassay, described here, with the SMN activity assay can be used to gain insight into the regulation of SMN protein expression and activity. These new assays may significantly add to the present methods available to study SMA.

## Conclusion

We describe a simple cell immunoassay to measure SMN levels in peripheral blood samples. This assay requires a small amount of patient material and can be done on a large number of samples simultaneously. The ability to measure SMN levels from peripheral blood samples provides an important snapshot of SMN expression that can be used as a surrogate outcome measure in ongoing and future SMA clinical trials.

## Competing interests

The author(s) declare that they have no competing interests.

## Authors' contributions

SJK carried out and participated in the development of the cell immunoassays, participated in the design and coordination of the study and drafted the manuscript. AKG, RFO and EO participated in the design of the study and in the development of the cell immunoassays. CJS collected and processed blood samples for the study. KHF participated in the design of the study. GD conceived of the study and participated in its design and coordination.

## Pre-publication history

The pre-publication history for this paper can be accessed here:
